# Supplementary material for: Tactile sensory coding and learning with bio-inspired optoelectronic spiking afferent nerves
Source: Nat Commun. 2020 Mar 13;11:1369. doi: 10.1038/s41467-020-15105-2 (PMC7070032; doi:10.1038/s41467-020-15105-2)
Supplement: Supplementary file 1 — Supplementary Information [file 41467_2020_15105_MOESM1_ESM.pdf]

Supplementary Information

Tactile sensory coding and learning with bioinspired optoelectronic spiking afferent nerves

Hongwei Tan*, Quanzheng Tao, Ishan Pande, Sayani Majumdar, Fu Liu, Yifan Zhou, Per O. Å. Persson, Johanna Rosen, Sebastiaan van Dijken*

*Correspondence to: hongwei.tan@aalto.fi (H. T.); sebastiaan.van.dijken@aalto.fi (S. v. D.)

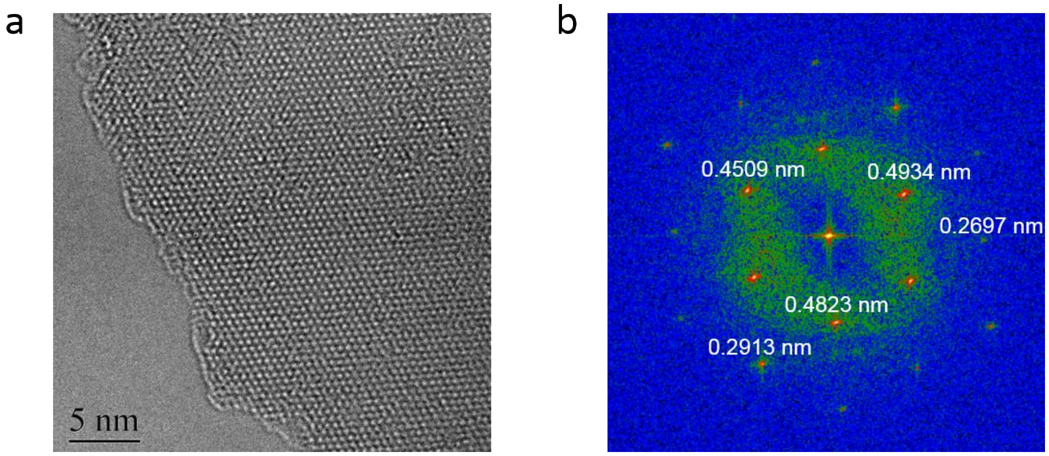

Supplementary Figure 1. Characterization of $Ti_3C_2T_x$ (MXene) nanosheet. a, Atomic resolution TEM image of a suspended $Ti_3C_2T_x$ (MXene) nanosheet from top view. **b**, Fast Fourier transform of (a). The distance marked in (b) corresponds to the lattice spacing in real space.

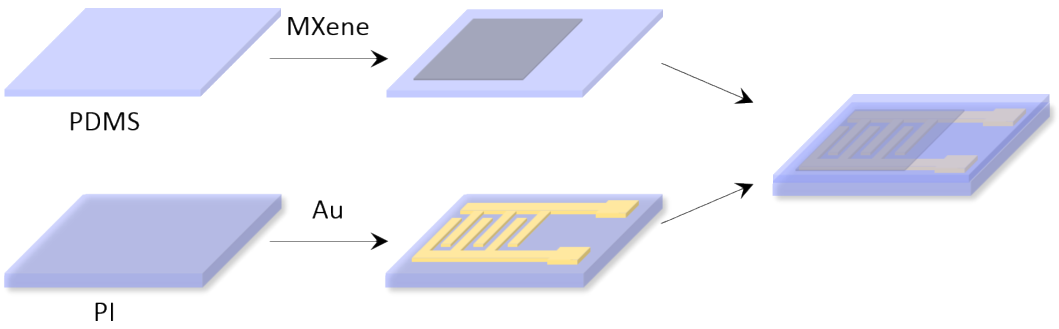

Supplementary Figure 2. Schematic of the fabrication process of the MXene-based pressure sensor.

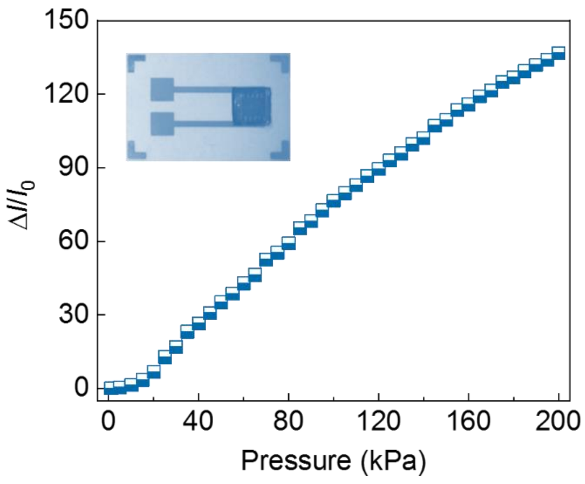

Supplementary Figure 3. Change of the current output from an MXene-based pressure sensor with increasing pressure. The insert shows an optical image of an MXene-based pressure sensor with working area of 5 mm × 5 mm.

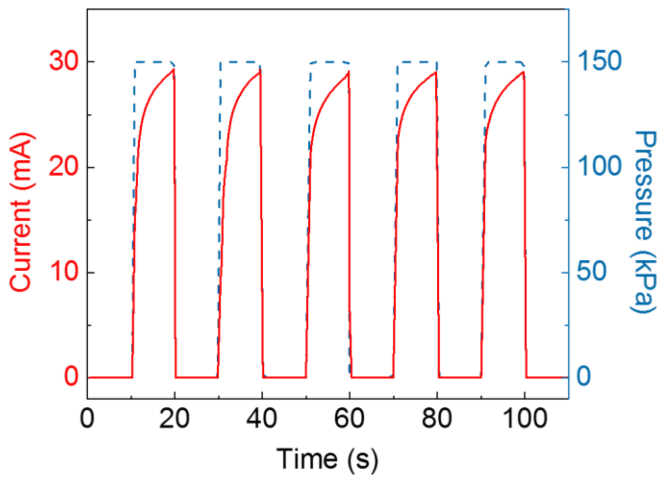

Supplementary Figure 4. Current response of an MXene-based sensor to repeated switching between on and off pressure states. The applied pressure is 150 kPa.

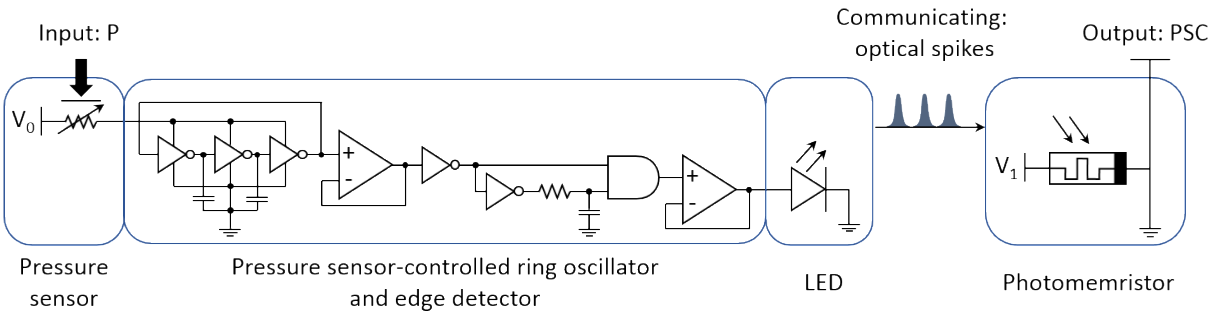

Supplementary Figure 5. Circuit diagram of the artificial afferent nerve system with optical communication. The ring oscillator converts the DC signal from the pressure sensor to an AC signal using an odd number of NOT gates. The oscillation frequency of the oscillator output depends on the voltage amplitude input (i.e. the pressure applied to the MXene sensor). The edge detector converts the AC signal to a digital signal (spikes) with identical pulse amplitude and width. The edge detector is made of two NOT gates, a resistor, a capacitor, and an AND gate. The resistor and capacitor are used to delay the input signal and modulate the output pulse width. The output of the edge detector is connected to an LED. The LED emits 375 nm light pulses with coded pressure information. The synaptic photomemristor integrates and processes the information coded in the light pulses.

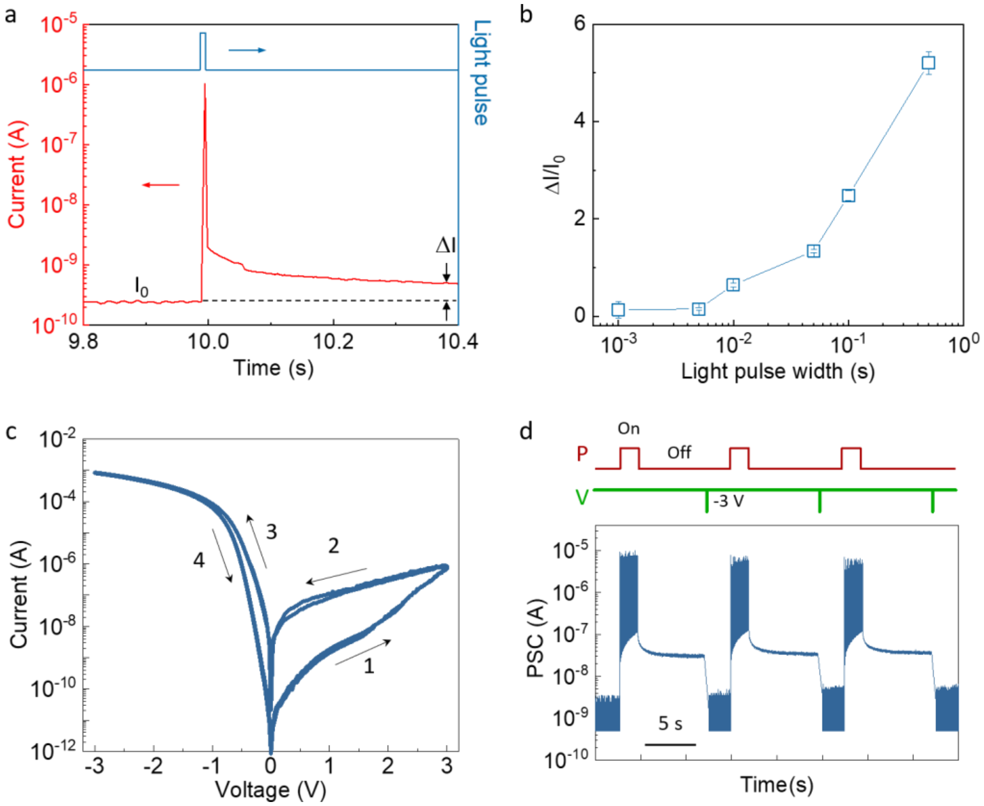

Supplementary Figure 6. Electrically erasable persistent photoconductivity of the photomemristor. a, Current response of a synaptic photomemristor to a single light pulse. **b**, Current change ratio in response to light pulses of different duration. The current change ratio decreases with decreasing pulse width. For a 1 ms light pulse, the current change ratio is about 13%. The error bars indicate variations during repeated measurements. **c**, Resistance switching cycles of the ITO/ZnO/Nb-STO optoelectronic memristor. **d**, Demonstration that the persistent photoconductivity effect of the ITO/ZnO/Nb-STO is quickly reset by the application of a negative voltage pulse. In the experiment, the PSC is reset to its initial value by a negative voltage pulse with amplitude –3 V and duration 0.5 ms.

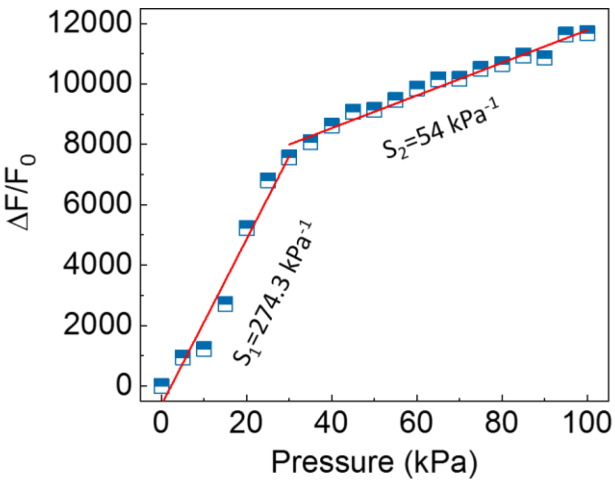

Supplementary Figure 7. Sensitivity of the tactile sensing system. The sensitivity is 274.3 kPa^{-1} for 0 – 30 kPa and 54 kPa^{-1} for 30 – 100 kPa. F_0 is the spiking rate without any input pressure and ΔF is the change in spiking rate when a pressure is applied. The sensitivity corresponds to the slope of the curve.

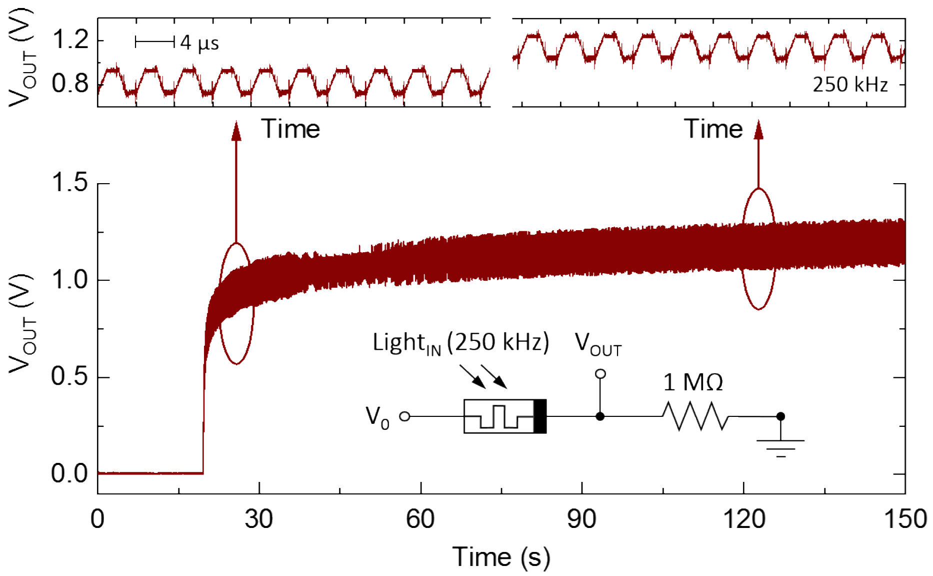

Supplementary Figure 8. Photoresponse of the synaptic photomemristor to light pulses with a frequency of 250 kHz. The duration of the light pulses is 2 µs. The insert shows the measurement circuit. The operation frequency of 250 kHz is the highest of any reported photomemristor thus far (Supplementary Table 1). Moreover, the measurement demonstrates good endurance over > 3×10^7 cycles. Based on these data, an energy consumption of ~ 4 pJ per light pulse is estimated.

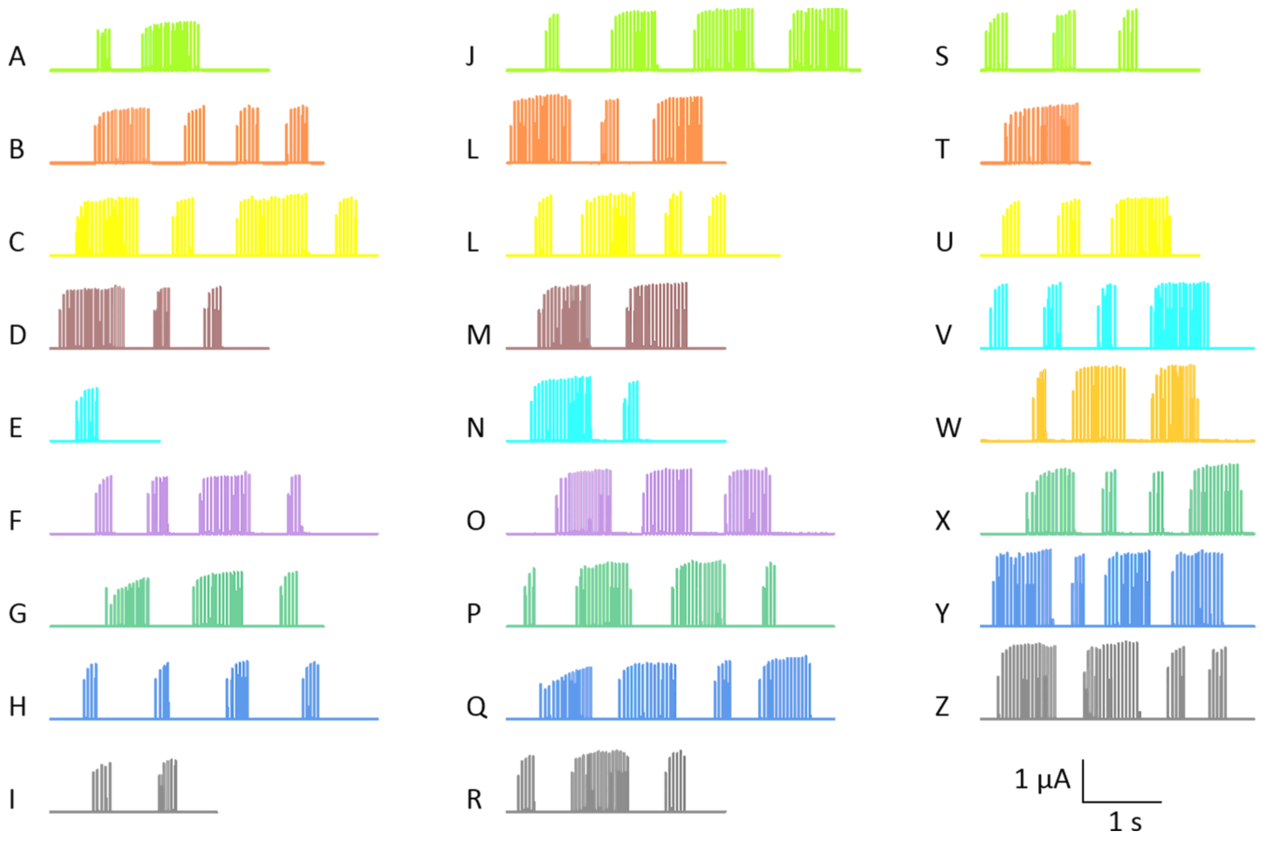

Supplementary Figure 9. PSCs in response to pressure information from Morse code letters of the alphabet. Spiking and quiescent timing encode the information. Short and long spiking times encode the dots and dashes in Morse code. Short and long quiescent times encode the spaces in Morse code letters and between letters.

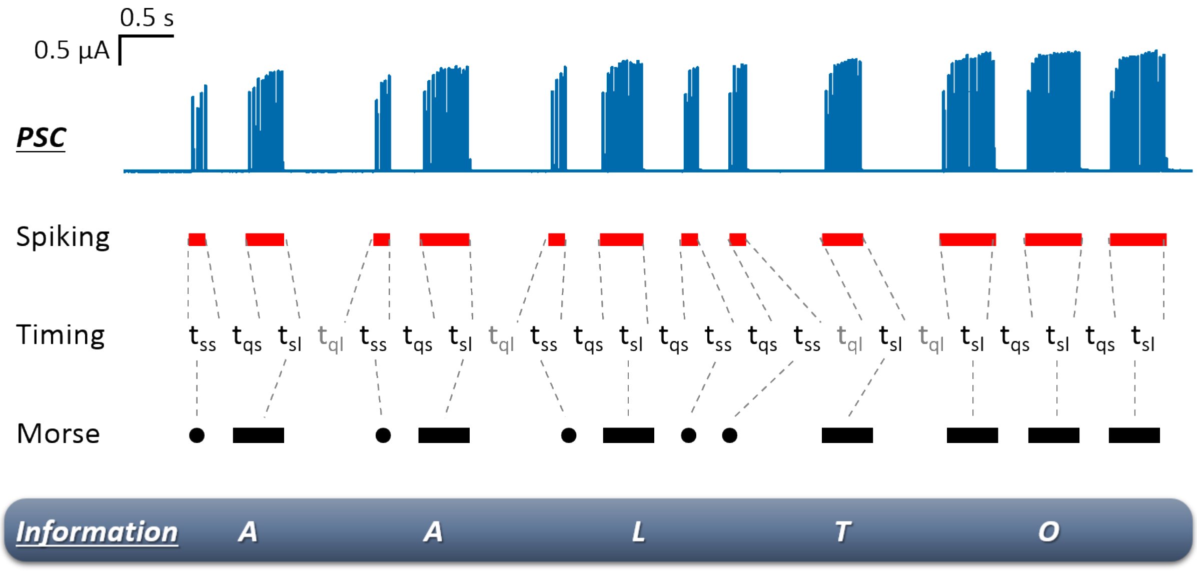

Supplementary Figure 10. Recognition of the word ‘AALTO’ in Morse code using tactile sensing. The temporal evolution of the PSC signal contains the input information.

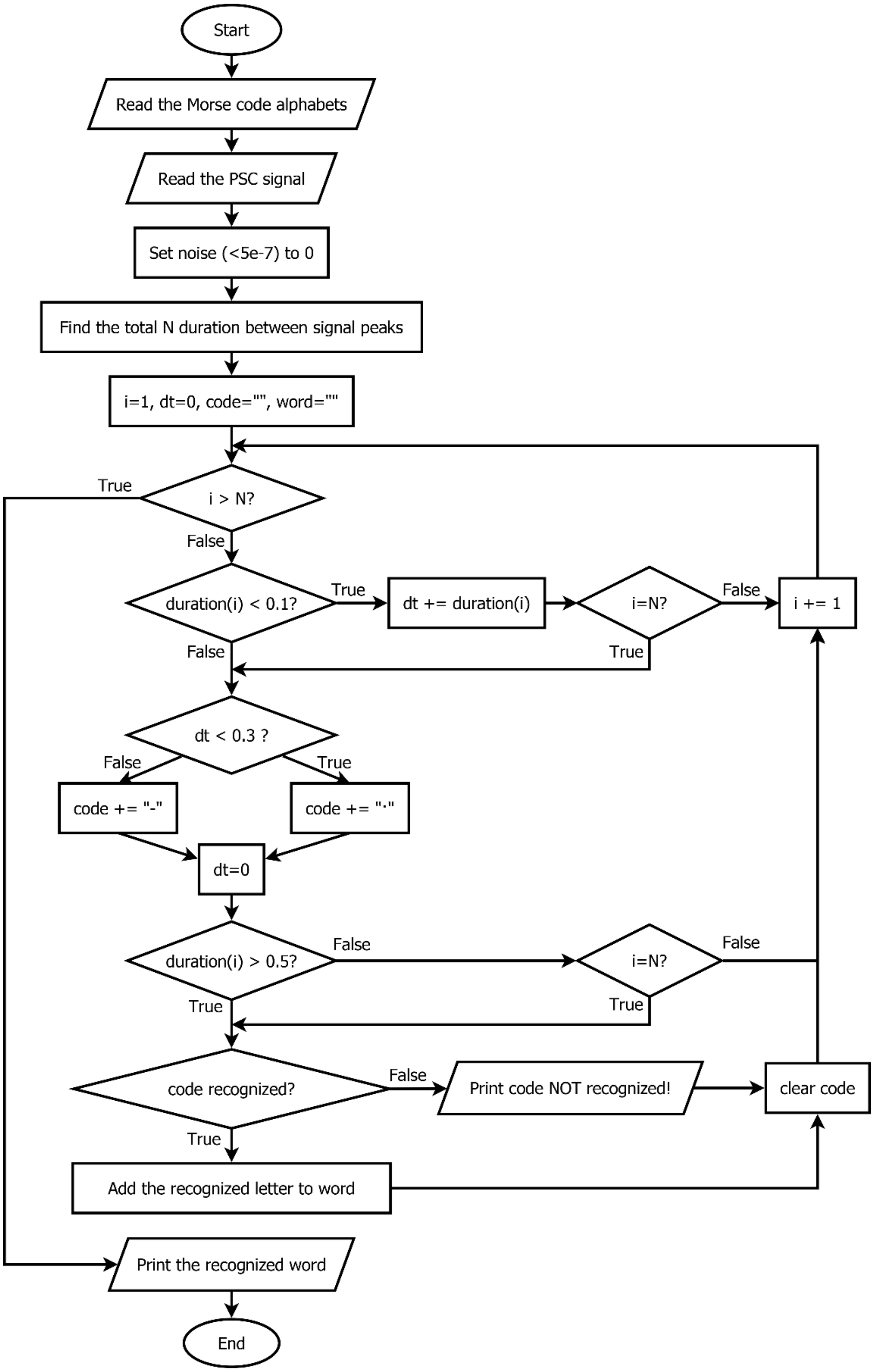

Supplementary Figure 11. Flow chart of the Morse code recognition process.

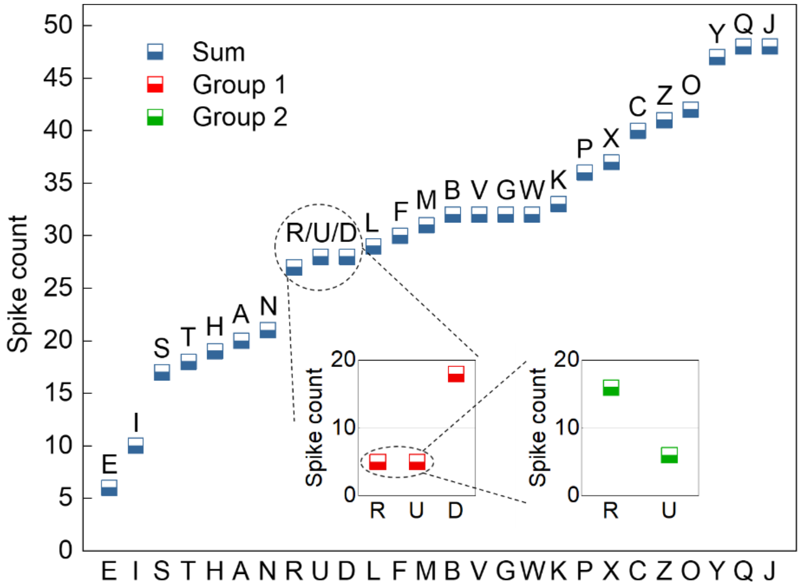

Supplementary Figure 12. Number of spikes produced by the optoelectronic spiking afferent nerve in response to pressure inputs from alphabet letters in Morse code. The output PSC signal of the optoelectronic memristor comprises several groups of spikes when Morse code characters are read (Figure S9). The total number of spikes defines each letter. If the spike count is similar (e.g. ‘R’, ‘U’, and ‘D’), the number of spikes in the first and second spiking group could be used to enhance the recognition accuracy (see inserts).

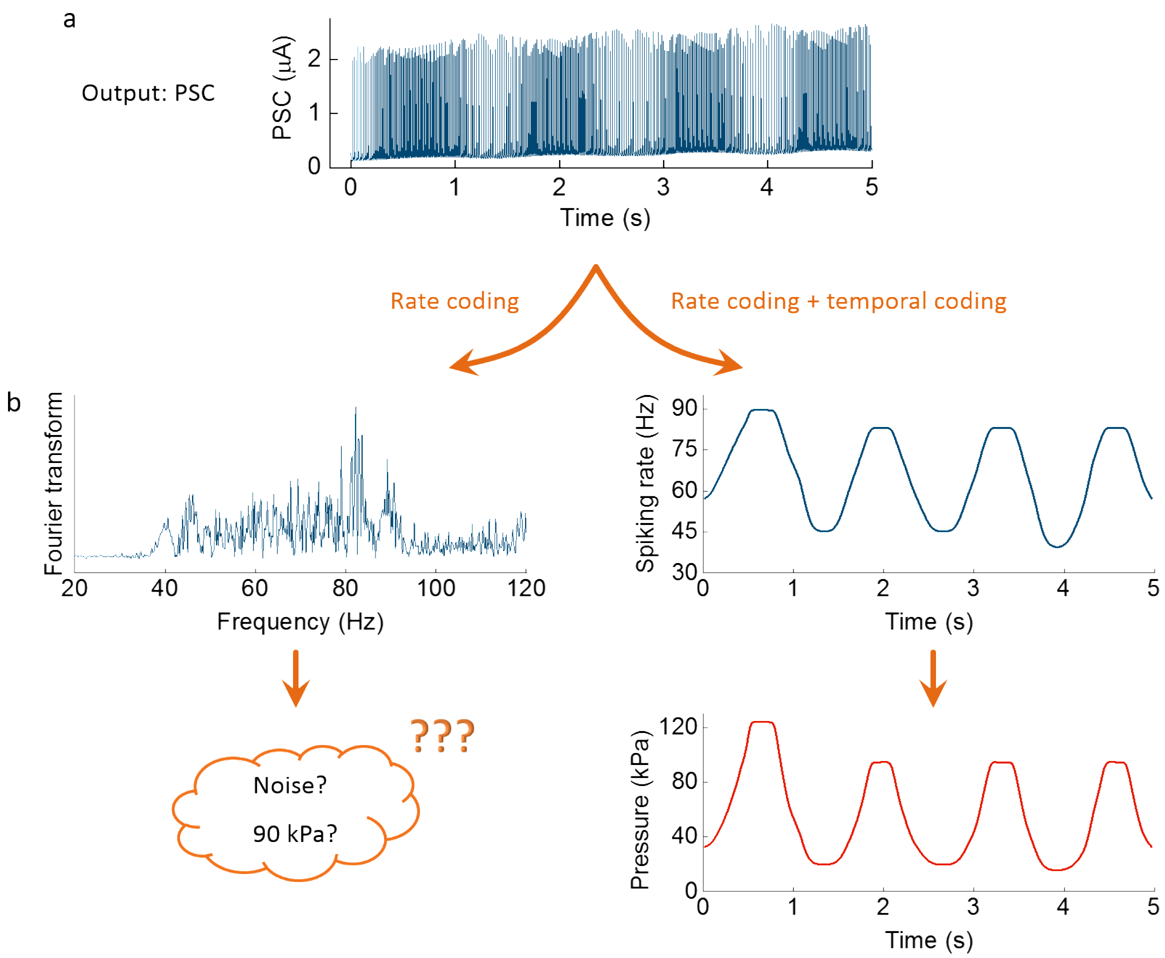

Supplementary Figure 13. Temporal coding with spiking rate fluctuation. a, PSC output for a fluctuating pressure input. **b**, Comparison of rate coding and a combination of rate coding and temporal coding. Rate coding works well when the input is a constant pressure. However, if the input pressure fluctuates, any information contained in those fluctuations will show up as noise in frequency spectra. In this case, a combination of rate and temporal coding can be used to correctly resolve the time evolution of pressure information.

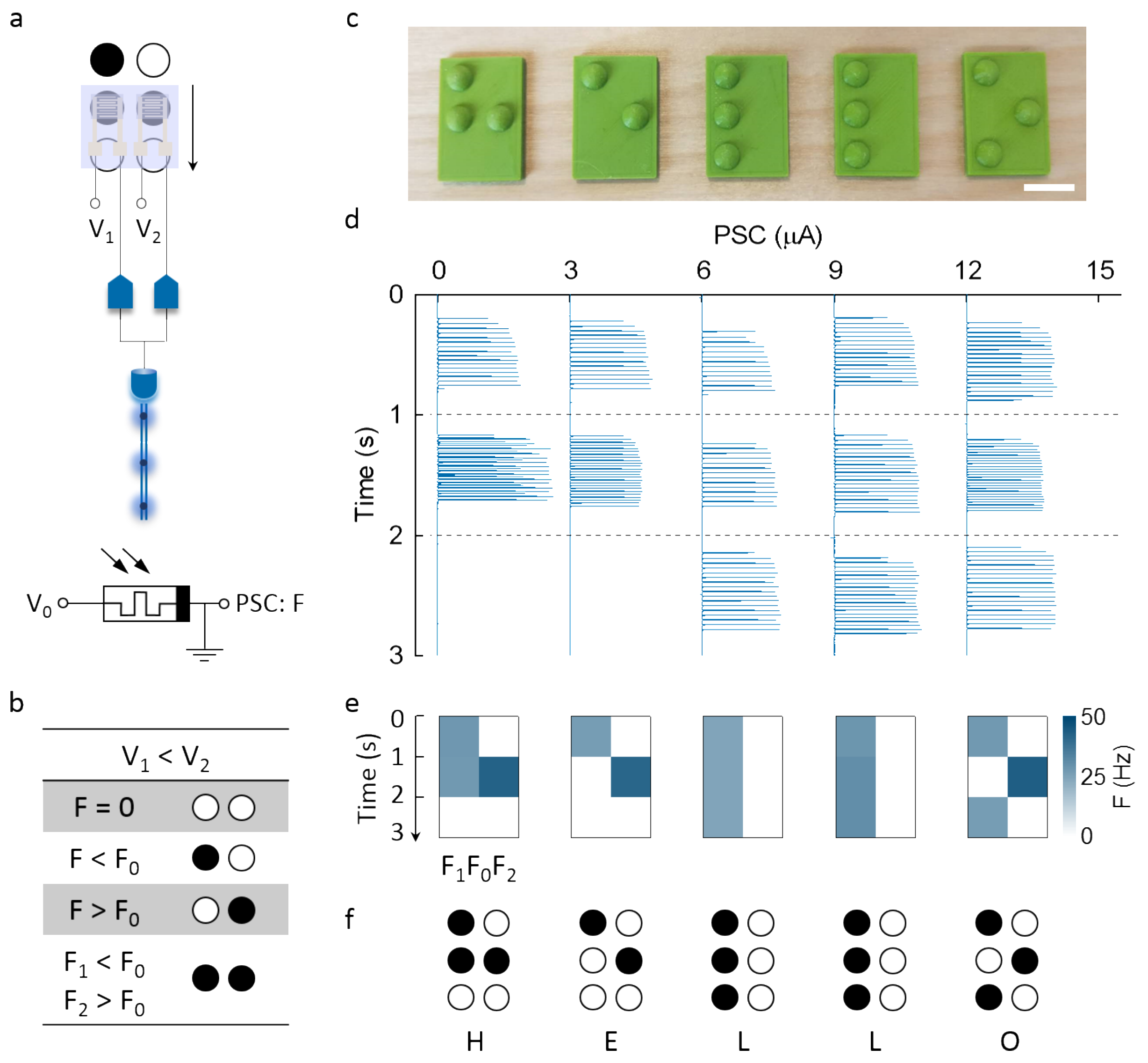

Supplementary Figure 14. Recognition of braille characters using tactile sensing. a, Schematic diagram of the braille recognition circuit. **b**, Decoding principle of braille information using PSCs with rate and temporal coding. Without convex pattern, there is no PSC output and, thus, the frequency (F) is zero. With convex patterns on the left and right, the frequency F_1 of the PSC is lower than the frequency F_2 of the PSC because V_1 is set to a lower value than V_2. F_0 is defined as intermediate frequency ($F_1 < F_0 < F_2$). **c**, Optical image of braille characters to be recognized in the test experiments. The scale bar corresponds to 1 cm. **d**, PSC outputs from the

synaptic photomemristor when reading the braille characters depicted in (c). **e**, Spiking rate and timing of the PSCs in (d). **f**, Recognized braille characters according to the principle in (b).

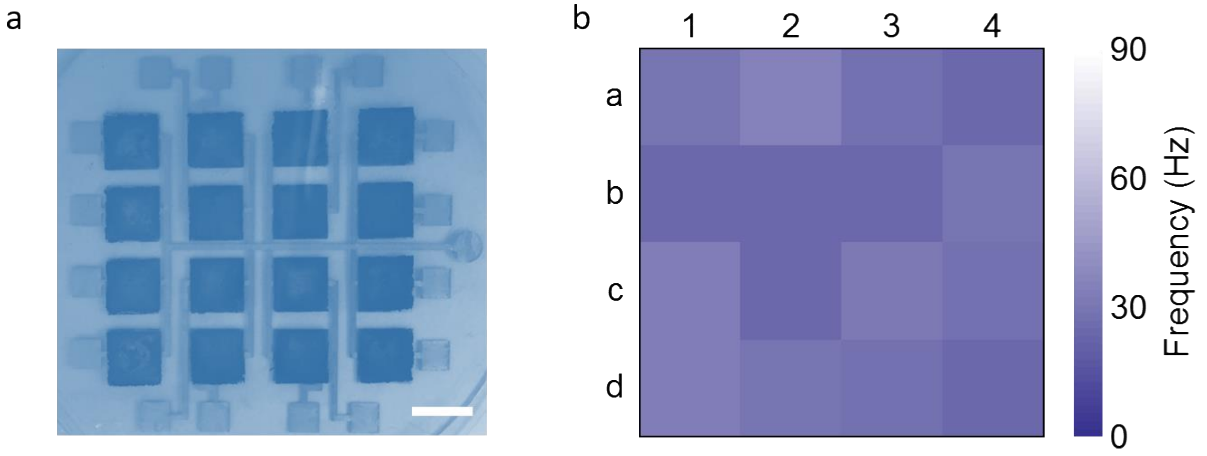

Supplementary Figure 15. Characterization of 4 × 4 sensor array. a, Optical image of a 4 × 4 sensor array. The scale bar corresponds to 5 mm. **b**, Frequency output of the 4 × 4 sensor array for a test pressure of 20 kPa, indicating uniformity.

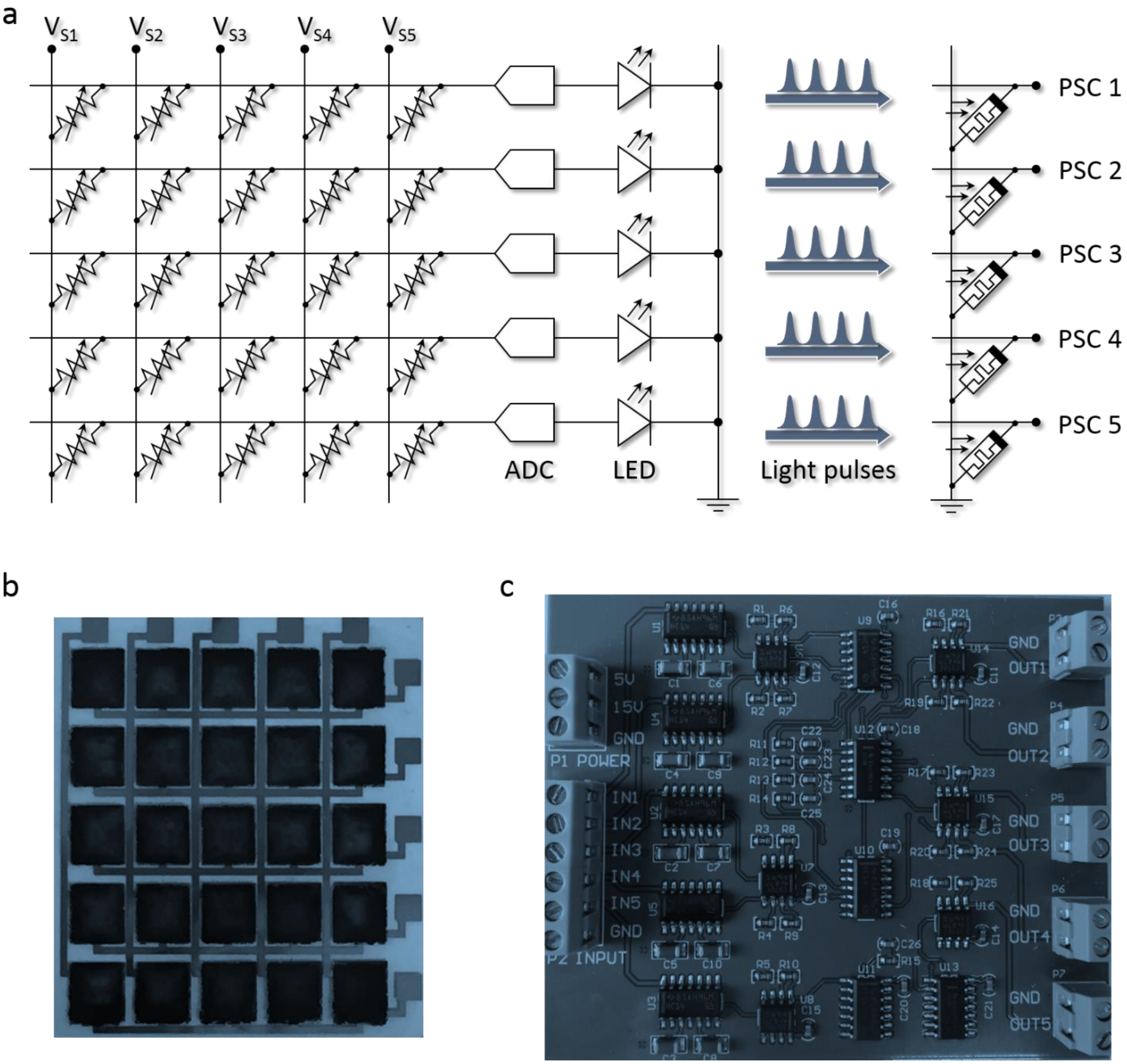

Supplementary Figure 16. Characterization of 5 × 5 sensor system. a, Circuit diagram of the dimensionality-reduced architecture for the recognition of handwritten letters of the alphabet. **b**,**c**, Sensor array (b) and printed circuit board (PCB) (c) that connects the sensor array and synaptic photomemristors for the recognition of handwritten letters of the alphabet.

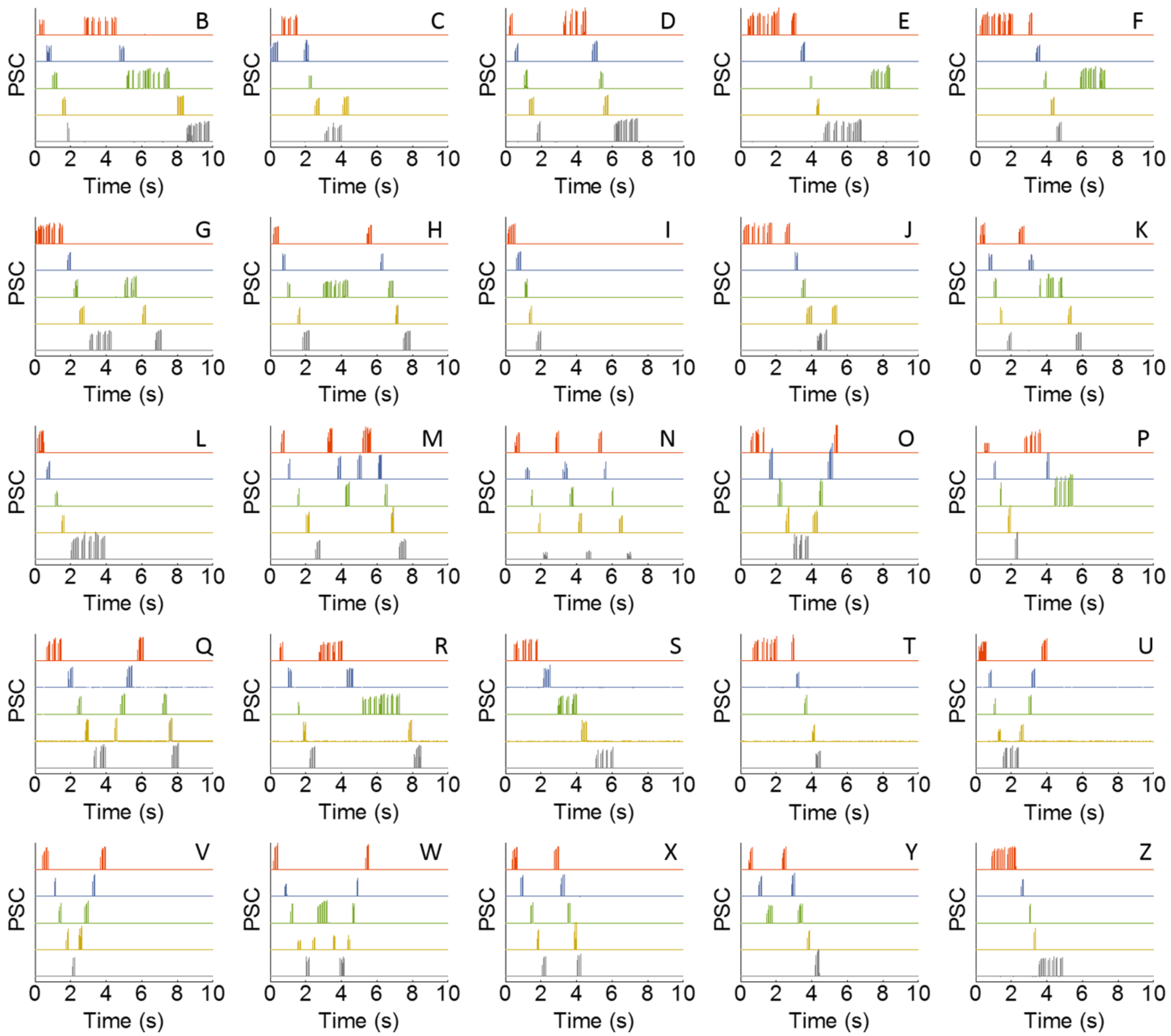

Supplementary Figure 17. PSC outputs from five synaptic photomemristors when writing the letters B-Z, implementing temporal coding of information with spiking duration and sequence. The initial feature dictionary $\overrightarrow{P_l}$ $(l = A, B, C, \ldots, Z)$ extracted from the PSCs in Supplementary Figure 16 is used as a database (Figure 4e) for subsequent recognition. With a given input $\overrightarrow{P_x}$, the recognition process is finding the nearest $\overrightarrow{P_l}$ to $\overrightarrow{P_x}$ in the 5D vector space, or the smallest $|\overrightarrow{P_x} - \overrightarrow{P_l}|$ $(l = A, B, C, \ldots, Z)$. After recognition, the $\overrightarrow{P_l}$ is updated as $(\overrightarrow{P_l}+\overrightarrow{P_x})$ / 2.

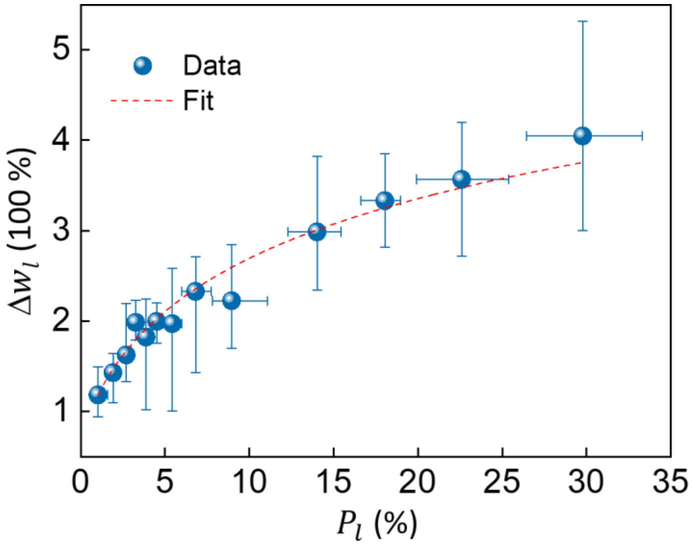

Supplementary Figure 18. Weight change as a function of spiking proportion measured after 20 cycles. Higher spiking proportions lead to larger weight changes, allowing weight changes to represent spiking proportions. Learning is based on a persistent photoconductivity effect in the photomemristor. The error bars indicate the minimum and maximum values. The red dashed line is a fit to the data.

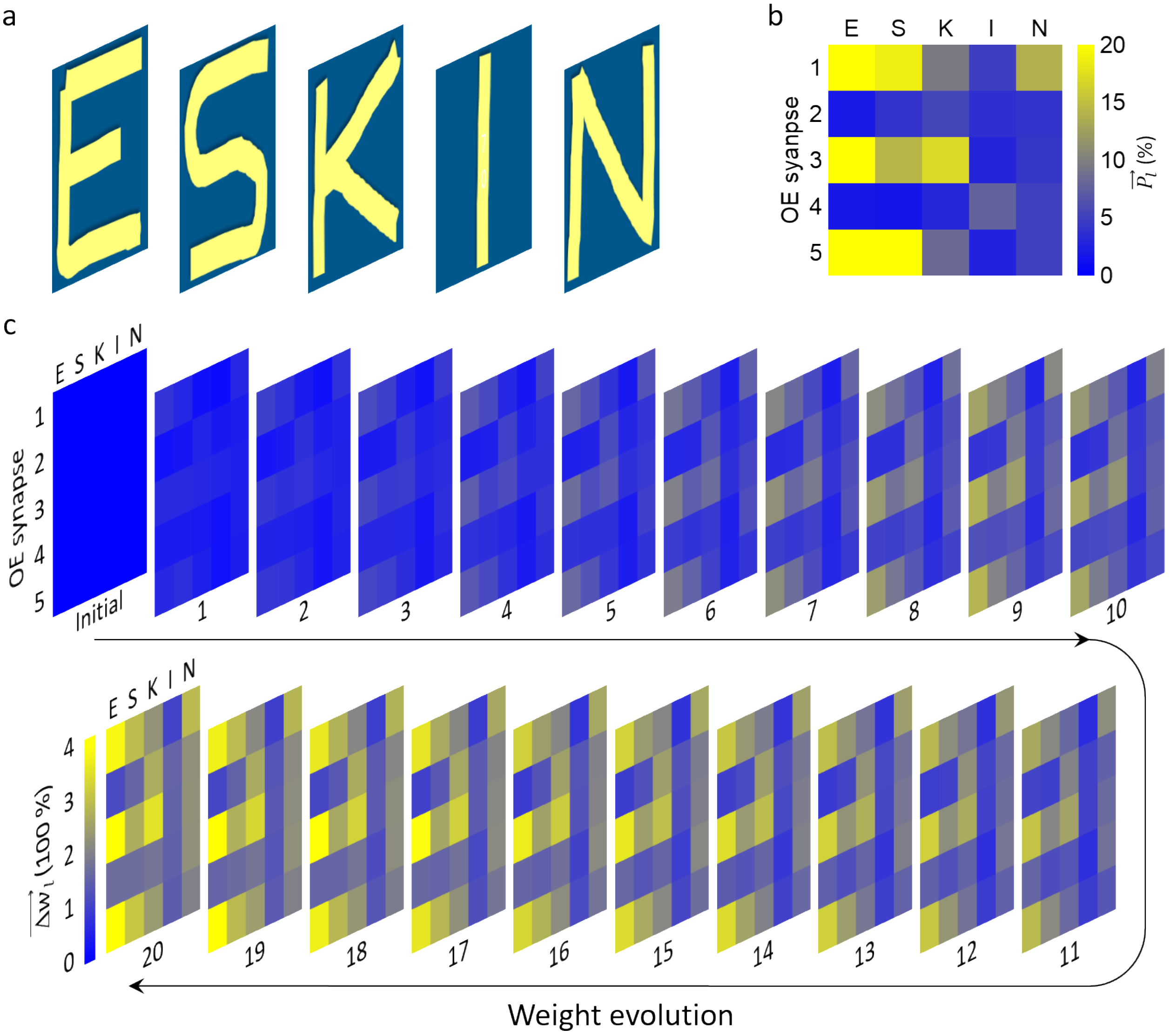

Supplementary Figure 19. Proof-of-concept demonstration of learning the handwritten word 'ESKIN'. a, Handwritten input of word 'ESKIN'. **b**, Spiking proportion ($\vec{P}_{\mathrm{ESKIN}}$) of the five synaptic photomemristors when writing 'ESKIN' on the 5 × 5 sensor array. **c**, Weight evolution during 20 epochs of the handwritten input 'ESKIN'.

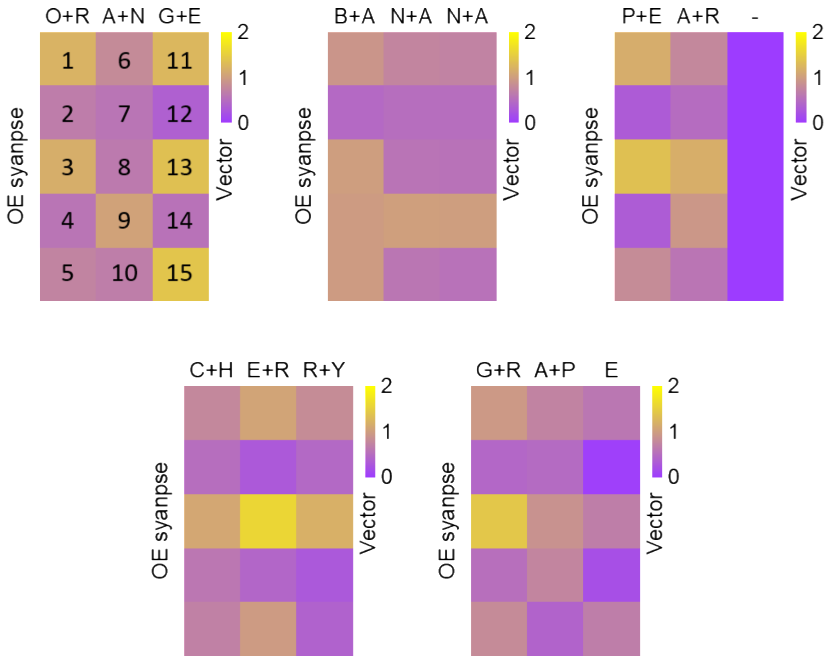

Supplementary Figure 20. Color map of dimensionality-reduced features for the words ‘ORANGE’, ‘BANANA’, ‘PEAR’, ‘CHERRY’, and ‘GRAPE’. The corresponding color map for ‘APPLE’ is shown in Figure 5b of the main manuscript. Reduction of the vector dimensionality is achieved by combining the pressure inputs from two subsequent handwritten letters (i.e. use of one synaptic photomemristor instead of two). Depending on the word, this reduces the dimensionality from twenty, twenty-five, or thirty to fifteen.

Supplementary Note 1. Code for Morse code recognition

```
SetDirectory[NotebookDirectory[]];
myPeaks[list_] :=
  Pick[list[[2 ;; -2]], Differences[Sign[Differences[list[[ ;; , 2]]]]], -2];
Letters = {"A", "B", "C", "D", "E", "F", "G", "H", "I", "J", "K", "L",
    "M", "N", "O", "P", "Q", "R", "S", "T", "U", "V", "W", "X", "Y", "Z"};
MorseCodes = {"•-", "-•••", "-•-•", "-••", "•", "••-•", "--•", "••••",
    "••", "•---", "-•-", "•-••", "--", "-•", "---", "•--•", "--•-",
    "•-•", "•••", "-", "••-", "•••-", "•--", "-••-", "-•--", "--••"};
Nletter = Length[Letters];
signal = Import["Morse-AALTO.dat", "Table"];
Do[If[signal[[it, 2]] < 0.1, signal[[it, 2]] = 0], {it, 1, Length[signal]}];
δts = Differences[myPeaks[signal][[ ;; , 1]]];
Nδt = Length[δts];
word = ""; wordcode = "";
error = False;
recog[code_] := If[Length[Position[MorseCodes, code]] == 0,
    {Print["Error: code ", code, " NOT recognized!"];
     error = True;},
    {word = word <> Letters[[Position[MorseCodes, code][[1, 1]]]];
     wordcode = wordcode <> code <> "    ";}];
i = 1;
dt = 0;
code = "";
While[i ≤ Nδt,
  If[δts[[i]] < 0.1,
   {dt += δts[[i]]},
   {If[dt < 0.3, code = code <> "•", code = code <> "-"];
    dt = 0;
    If[δts[[i]] > 0.5, {recog[code]; code = "";}]}];
  If[i == Nδt,
   {If[dt < 0.3, code = code <> "•", code = code <> "-"];
    dt = 0; recog[code]; code = "";}
  ];
  i += 1;
 ];
If[error, Print["There is unrecognizable code!"]];
Print["The recognized word is: ", word]
Print["The recognizable codes are: " wordcode]
```

The boundary values used in this code (0.1, 0.3, and 0.5) are learned from PSCs induced by pressure inputs with Morse code information. Here, 0.1 is the boundary between spiking and non-spiking, 0.3 is the boundary between a dot and dash, and 0.5 is the boundary between a dash and the space between two letters.

Supplementary Note 2. Code for handwriting recognition

```
SetDirectory[NotebookDirectory[]];
Letters = {"A", "B", "C", "D", "E", "F", "G", "H", "I", "J", "K",
   "L", "M", "N", "O", "P", "Q", "R", "S", "T", "U", "V", "W", "X", "Y", "Z"};
Nletter = Length[Letters];
A2Zi = {};
Do[AppendTo[A2Zi, Import[Letters[[idx]] <> ".dat", "Table"]], {idx, 1, Nletter}];
Nround = Length[A2Zi[[1]]];
check[iround_, iletter_] := Module[{coord, norms, pos},
   coord = A2Zi[[iletter, iround]];
   norms = Norm[A2Z0[[#]] - coord] & /@ Range[Nletter];
   pos = Position[norms, Min[norms]][[1, 1]];
   pos == iletter
  ];
A2Ztraning[Ntraining_] := Module[{A2Ztemp},
   A2Ztemp = A2Zi[[ ;; , 1,  ;;]];
   Do[A2Ztemp += A2Zi[[ ;; , i,  ;;]];, {i, 2, Ntraining}];
   A2Ztemp / Ntraining
  ];
Ntrain = 10;
iTrueFalse = {};
Do[
  A2Z0 = A2Ztraning[itraining];
  true = 0; false = 0;
  Do[
   Do[
     If[check[iround, iletter], true += 1, false += 1];
     , {iletter, 1, Nletter}];
   , {iround, Ntrain + 1, Nround}];
  AppendTo[iTrueFalse, {itraining, 1. true/(true + false), 1. false/(true + false)}];
  , {itraining, 1, Ntrain}];
iTrueFalse
ListLinePlot[{iTrueFalse[[ ;; , {1, 2}]], iTrueFalse[[ ;; , {1, 3}]]},
 PlotMarkers → Automatic, PlotRange → {0, 1}, Frame → True,
 FrameLabel → {"Traning number", "Accuracy"},
 PlotLegends → {"Correct Rate", "Wrong rate"}]
```

During the first learning cycle, handwritten letters are represented by the spiking proportions of five memristors forming different vectors $\vec{P}$ for each letter. The 26 vectors create a dictionary for learning and recognition (Figure 4e).

Supplementary Table 1. Summary of typical optoelectronic synapses.

Material	Structure	Spiking/ PPC[1]	Response time	On/off	Read	Working speed	Endurance	Power (/spike)	Ref.
IGZO	Transistor	PPC	> 1 s	-	10 V	1 Hz	-	25 nJ	[1]
CsPbBr3 QDs	Transistor	PPC	> 1 s	~ 20	-20 V	1 Hz	-	700 nJ	[2]
ITO/Nb:STO	Memristor	PPC	0.5 s	< 3	50 mV	1 Hz	-	~ 875 pJ	[3]
MoO_x	Memristor	PPC	0.2 s	~ 40	0.1 V	2.5 Hz	-	~ 66 pJ	[4]
**ZnO/Nb:STO**	**Memristor**	**Spiking & PPC**	**2 μs**	**10^4**	**1 V**	**250 kHz**	**> 3 × 10^7**	**~ 4 - 1000 pJ**	**This work**

[1]PPC: persistent photoconductivity

Supplementary Table 2. Summary of typical artificial afferent nerve systems.

Sense	Communicate	Process	Neural coding	Learning	Memory	Sensing		Processing		Ref.
						Working range	Sensitivity (kPa^{-1})	Power (/spike)	Endurance	
Pressure-sensing OFET	DC[1]	Signal-processing OFET	Amplitude	-	-	40 - 100 Pa	8 - 192	60 nJ	-	[5]
CNTs/PDMS pyramid	DC	Synaptic transistor	Amplitude	Supervised learning	✓	0.1 - 1 kPa	0.8 - 80	1.5 μJ	-	[6]
Au/PDMS Pyramid	DC	Nafin-memristor	Amplitude	Supervised learning	-	1 - 50 kPa	6.7×10^7<br>3.8×10^5	10 - 200 pJ	$> 10^4$	[7]
P(VDF-TrFE)	DC	Gel-gated graphene transistor	Amplitude	-	-	0 – 0.9 % (strain)	193	Self-powered	-	[8]
Tactile glove	DC	CNN[2]	Amplitude	Supervised learning	✓	0.02 - 0.5 N	-	-	-	[9]
CNTs/pyramid-structured elastomer	Voltage spikes	Synaptic transistor	Rate coding	-	-	1 - 80 kPa	-	30 nJ	-	[10]
**MXene-based pressure sensor**	**Optical spikes**	**Synaptic photomemristor**	**Rate coding**<br>**Temporal coding**<br>**Feature extraction**	**Supervised learning**<br>**Feature learning**	✓	**1-100 kPa**	**274**<br>**54**	**4 - 1000 pJ**	**$> 3 \times 10^7$**	**This work**

[1]DC: direct current; [2]CNN: convolutional neural network

Supplementary References

1. Lee, M. et al. Brain-inspired photonic neuromorphic devices using photodynamic amorphous oxide semiconductors and their persistent photoconductivity. *Adv. Mater.* **29**, 1700951 (2017).
2. Wang, Y. et al. Photonic synapses based on inorganic perovskite quantum dots for neuromorphic computing. *Adv. Mater.* **30**, 1802883 (2018).
3. Gao, S. et al. An oxide Schottky junction artificial optoelectronic synapse. *ACS Nano* **13**, 2634-2642 (2019).
4. Zhou, F. et al. Optoelectronic resistive random access memory for neuromorphic vision sensors. *Nat. Nanotechnol.* **14**, 776-782 (2019).
5. Zang, Y., Shen, H., Huang, D., Di, C.-A. & Zhu, D. A dual-organic-transistor-based tactile-perception system with signal-processing functionality. *Adv. Mater.* **29**, 1606088 (2017).
6. Wan, C. et al. An artificial sensory neuron with tactile perceptual learning. *Adv. Mater.* **30**, 1801291 (2018).
7. Zhang, C. et al. Bioinspired artificial sensory nerve based on nafion memristor. *Adv. Funct. Mater.* **29**, 1808783 (2019).
8. Chen, Y. et al. Piezotronic graphene artificial sensory synapse. *Adv. Funct. Mater.* 1900959 (2019).
9. Sundaram, S. et al. Learning the signatures of the human grasp using a scalable tactile glove. *Nature* **569**, 698-702 (2019).
10. Kim, Y. et al. A bioinspired flexible organic artificial afferent nerve. *Science* **360**, 998-1003 (2018).
